# Recurrent Peristomal Variceal Hemorrhage Secondary to Portal Hypertension: A Diagnostic Pitfall in Metastatic Rectal Cancer

**DOI:** 10.7759/cureus.113328

**Published:** 2026-07-24

**Authors:** Hamza A Ashfaq, Ashok Sah, Nizar Alnabahneh, Benjamin Frimodig, Ahmid Al-Shawk

**Affiliations:** 1 Department of Internal Medicine, Wayne State University, Detroit, USA; 2 Department of Radiology, Wayne State University, Detroit, USA; 3 School of Medicine, Wayne State University, Detroit, USA

**Keywords:** computed tomography angiography, ectopic varices, gastrointestinal hemorrhage, ostomy bleeding, peristomal varices, portal hypertension, portal venous collaterals, rectal adenocarcinoma

## Abstract

Peristomal varices are an uncommon but important cause of recurrent bleeding in patients with portal hypertension and enterostomies. They may be mistaken for local trauma, mucosal irritation, superficial abdominal wall vessel bleeding, or malignant bowel invasion, delaying definitive management. We present a 72-year-old female patient with stage IV rectal adenocarcinoma metastatic to the liver who developed recurrent stomal hemorrhage initially attributed to small bowel bleeding. Repeat multidisciplinary review of computed tomography angiography demonstrated abnormal peristomal vasculature consistent with portal venous collaterals, favoring peristomal varices as the bleeding source. Recognition of the true bleeding source redirected management toward treatment of portal hypertension with octreotide and non-selective beta blockade and facilitated short-term hemostatic control. This case highlights the importance of considering peristomal varices in patients with recurrent ostomy bleeding and portal hypertension, particularly when standard gastrointestinal bleeding evaluations are unrevealing. Careful reassessment of existing imaging may identify portal venous collaterals and substantially alter management.

## Introduction

Ectopic varices account for a minority of variceal bleeding episodes but can produce substantial morbidity and mortality [1,2]. Peristomal varices develop at the mucocutaneous junction of an ileostomy or colostomy when portal hypertension promotes the formation of portal venous collaterals between mesenteric venous tributaries and systemic veins of the abdominal wall [1-3]. They are an important but frequently underrecognized cause of recurrent stomal bleeding in patients with portal hypertension and enterostomies. Bleeding may be intermittent, recurrent, and difficult to localize, particularly when obscured by ostomy appliances or clot burden, and is often attributed to mucosal trauma, granulation tissue, malignant invasion, or intraluminal gastrointestinal hemorrhage rather than ectopic varices [3-5]. Clinical manifestations range from chronic occult blood loss with iron deficiency anemia to life-threatening hemorrhage requiring urgent resuscitation [3,6].

Although population-level data are limited, peristomal variceal bleeding has been reported in up to 27% of patients with portal hypertension and enterostomies within five years of ostomy creation, though the true incidence is likely underestimated because of underrecognition and competing diagnoses [4,5]. A Japanese nationwide survey further highlights the rarity of this condition, reporting that stomal varices comprised only 2% of all ectopic varices [7].

Computed tomographic angiography (CTA), Doppler ultrasound, and portal venography are valuable for identifying portal venous collateral anatomy and guiding management [1-4]. Treatment is directed toward both hemorrhage control and reduction of portal hypertension. Definitive therapies include transjugular intrahepatic portosystemic shunt (TIPS), selective embolization, and percutaneous sclerotherapy, although treatment must be individualized according to vascular anatomy, liver reserve, comorbidities, and procedural risk [2,5,8-12].

We present a patient with metastatic rectal adenocarcinoma whose recurrent ostomy hemorrhage was initially attributed to intraluminal gastrointestinal bleeding until multidisciplinary reinterpretation of vascular imaging identified peristomal varices as the true bleeding source. The diagnosis redirected management toward treatment of portal hypertension in a patient who was not a candidate for definitive portal decompression or targeted endovascular therapy.

## Case presentation

A 72-year-old female patient with rectal adenocarcinoma previously treated with radiation and multiple chemotherapy regimens, status post loop colostomy, and with a history of presacral fistula/abscess with concern for osteomyelitis presented with relapsing-remitting gastrointestinal bleeding after noticing a large volume of coagulated blood in her ostomy bag with symptoms of acute anemia. Admission laboratory studies, summarized in Table 1, demonstrated severe acute blood loss anemia (hemoglobin 3.9 g/dL), hypoalbuminemia, mild lactic acidosis, preserved renal function, and no coagulopathy. The patient was admitted to the medical intensive care unit for close hemodynamic monitoring.

**Figure 1:**
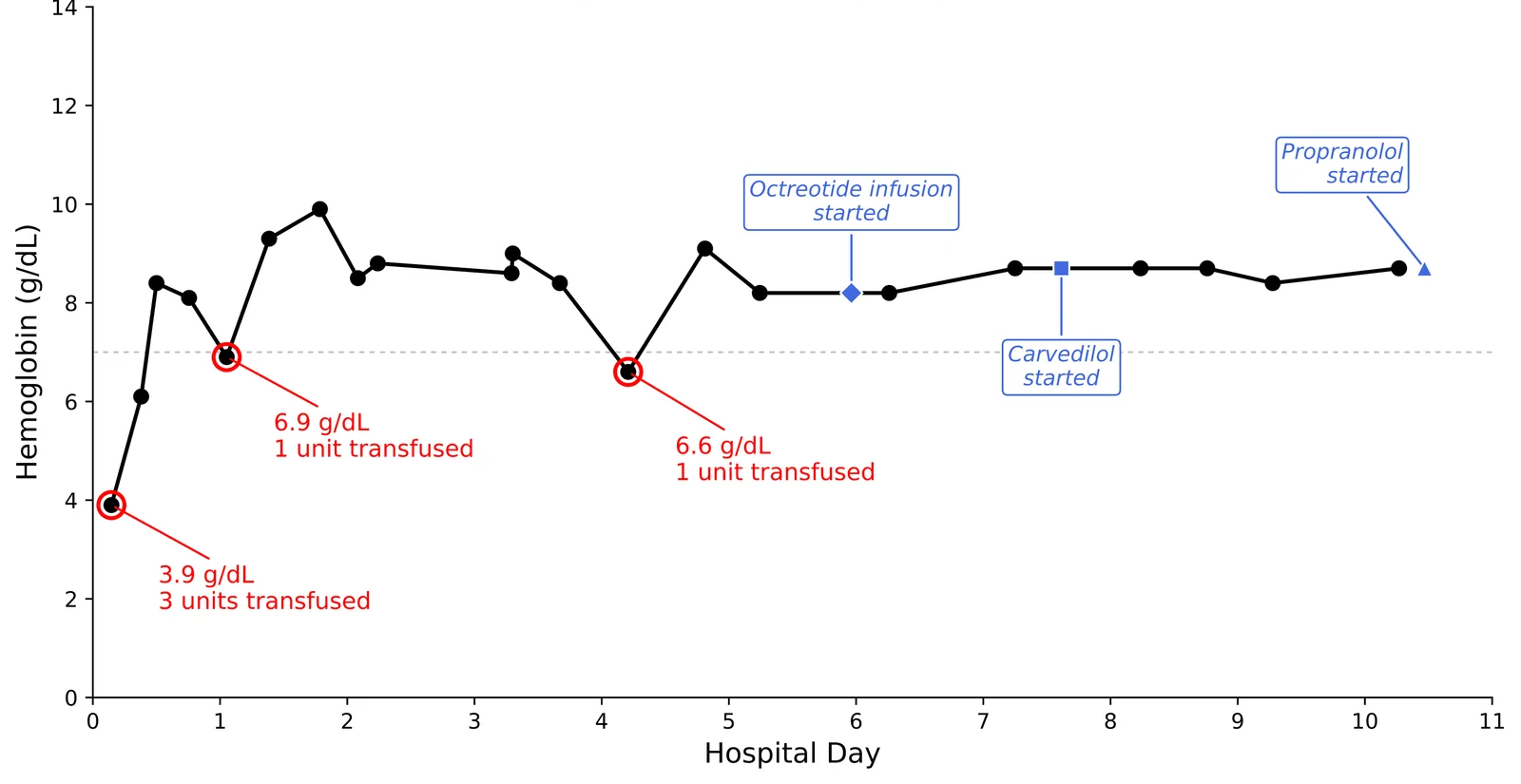
Hemoglobin trend during hospitalization. Hemoglobin measurements are plotted using the actual laboratory collection times, with the x-axis representing hospital day (Day 0 = midnight on the day of admission). Black circles represent individual hemoglobin measurements, and the dashed horizontal line denotes the transfusion threshold of 7 g/dL. Red outlined circles and annotations identify hemoglobin values that prompted packed red blood cell transfusions (3 units at 3.9 g/dL, 1 unit at 6.9 g/dL, and 1 unit at 6.6 g/dL). Blue markers indicate initiation of medical therapy: octreotide infusion (diamond), carvedilol (square), and propranolol (triangle).

The patient was initially transfused with three units of packed red blood cells (pRBCs), and hemoglobin improved from 3.9 g/dL to 8.4 g/dL (Figure 1). Initial CT abdomen/pelvis with and without contrast showed no active gastrointestinal bleeding but demonstrated cirrhotic liver morphology, hepatic lesions concerning for metastases, pulmonary nodules, extensive ascites, diffuse anasarca, bilateral hydronephrosis (right greater than left), and a presacral/perirectal abscess.

On the second day of hospitalization, the patient was deemed to be hemodynamically stable enough for transfer to the general medical floor. Upon arrival to the medical floor, further workup for gastrointestinal bleeding was initiated. Four months earlier, she had been hospitalized in another health system for severe blood loss anemia requiring revocation of hospice status and transfusion of blood products. No bleeding source was identified, though a lower gastrointestinal source was suspected. In the interim, leading up to the present admission, she remained iron-deficient and required additional transfusions and iron supplementation.

Persistent anemia required additional pRBC transfusions, including one unit on day 2 for a hemoglobin of 6.9 g/dL. The patient had an episode of grossly bloody, coagulated ostomy output on day 3, which heightened concern for an unrecognized venous bleeding source. A further decline in hemoglobin to 6.6 g/dL on day 5 required an additional unit of pRBCs. Iron studies were obtained after transfusion and should therefore be interpreted with caution (Table 1). Iron infusions were initiated after calculating iron deficit according to the Ganzoni equation for iron deficiency.

**Table 1:** Key laboratory findings on admission and following initial transfusion of packed red blood cells

	Admission Values	Post-Transfusion Iron Studies	Reference Range
Hemoglobin	3.9 g/dL	-	11.5-15.1 g/dL
Hematocrit	13.6%	-	34.4-44.2%
Iron Saturation	-	5%	20-50%
Iron	-	12 µg/dL	50-212 µg/dL
Ferritin	-	80.9 ng/mL	11-306.8 ng/mL
Total Iron Binding Capacity	-	241 µg/dL	250-450 µg/dL
Albumin	2.4 g/dL	-	3.5-5.7 g/dL
Creatinine	0.64 mg/dL	-	0.6-1.2 mg/dL
Lactic Acid	2.1 mmol/L	-	0.4-2.0 mmol/L
Prothrombin Time	11.2 seconds	-	9.4-11.7 seconds
International Normalized Ratio	1.07	-	0.89-1.12

Physical examination revealed abdominal striae and shifting dullness without abdominal tenderness, jaundice, scleral icterus, or caput medusae. The stoma appeared normal without evidence of trauma, active bleeding, abnormal vasculature, or malformation. The patient was intermittently hypotensive, with a nadir blood pressure of 95/49 mmHg, but subsequently stabilized with mean arterial pressures greater than 65 mmHg.

**Figure 2:**
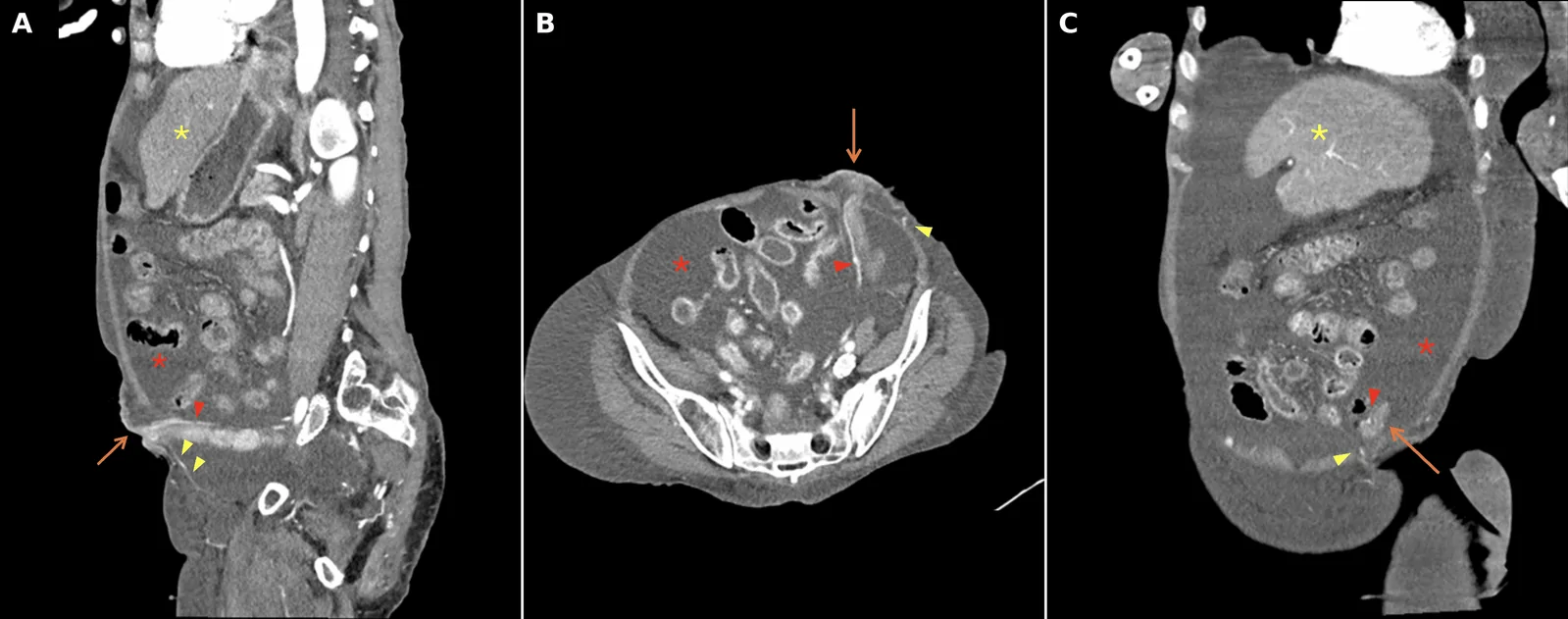
Sagittal, axial, and coronal CT images (A, B, and C, respectively) demonstrate a loop colostomy in the left lower quadrant (orange arrow). Engorged small veins along the ostomy conduit (red arrowheads) communicate with systemic veins within the abdominal wall near the ostomy exit (yellow arrowheads), forming peristomal varices. Additional findings include large-volume ascites (red asterisks) and cirrhotic liver morphology (yellow asterisks). Image quality was limited by motion and upper-extremity beam-hardening artifacts related to the patient’s clinical condition.

The gastroenterology service was consulted, and they recommended obtaining another CT angiography of the abdomen and pelvis. Repeat CT abdomen/pelvis again showed no active luminal bleeding but identified small peristomal varices adjacent to the left lower quadrant ostomy, which were considered the most likely bleeding source (Figure 2). One year prior, the gastroenterology team had performed esophagogastroduodenoscopy and colonoscopy through the colostomy, with evaluation of both the proximal and distal bowel, for a similar presentation of blood loss anemia without an identifiable bleeding source. Endoscopic evaluation demonstrated no active bleeding from the upper gastrointestinal tract, loop colostomy, or stoma. Blood clots were present throughout the colon without an identifiable active bleeding source. An erythematous, obstructing rectal mass with signs of recent bleeding was noted and was considered a potential source of hemorrhage. During that same hospitalization, the serum-ascites albumin gradient (SAAG) was 1.8. Although the patient presented with severe blood loss anemia with an initial hemoglobin of 3.9 g/dL, repeat CT angiography demonstrated no active luminal extravasation, and no blood per rectum was observed during the current hospitalization. Furthermore, endoscopic evaluation performed one year earlier for a similar presentation had not identified a definitive luminal bleeding source, while current imaging demonstrated peristomal varices as the suspected source of hemorrhage. Given these findings, repeat endoscopy was considered unlikely to identify an endoscopically treatable luminal source or provide additional therapeutic benefit and was therefore not pursued.

Multidisciplinary review with gastroenterology and radiology favored portal venous collaterals rather than superficial inferior epigastric vessels, supporting the diagnosis of peristomal varices. Tumor invasion, abnormal peristomal vascularity, and small bowel active luminal bleeding were excluded based on imaging characteristics on CT and venogram, which demonstrated engorged venous structures consistent with varices rather than infiltrative, ischemic, or actively hemorrhaging changes, with no evidence of active extravasation on repeat CTA. Physical examination yielded low suspicion of granulation tissue, mucosal trauma, or ischemia. Furthermore, portal hypertension was suspected based on cirrhotic liver morphology, ascites, and a previously documented SAAG of 1.8.

The gastroenterology team recommended interventional radiology consultation to assess the feasibility of treating the portal venous collaterals. The patient was not considered a suitable candidate for transjugular intrahepatic portosystemic shunt (TIPS) placement because of her overall poor clinical status and unfavorable risk-benefit profile. Although her MELD-Na (Model for End-Stage Liver Disease-Sodium) score of 7 and Child-Pugh Class B cirrhosis did not independently preclude TIPS, she had advanced metastatic malignancy with hepatic involvement, poor functional status (Eastern Cooperative Oncology Group (ECOG) 3), low estimated overall survival, and hemodynamic instability with hypotension. In the setting of advanced, life-limiting malignancy and substantial procedural risk, the potential benefits of portal decompression were considered unlikely to outweigh the risks of TIPS placement. Therefore, a less invasive, targeted approach to control the peristomal variceal hemorrhage was pursued.

Targeted interventional management of the peristomal variceal complex with sodium tetradecyl sulfate (STS) sclerotherapy was also considered. Venography was performed in preparation for possible intervention. The portal venogram confirmed portal venous collaterals communicating with the peristomal veins but demonstrated no active extravasation or significant communication between the recanalized umbilical vein and the left lower quadrant loop colostomy. Direct ultrasound-guided access to the multifocal peristomal varices was unsuccessful because of their small caliber. A retrograde portal venous approach was considered; however, after multidisciplinary discussion with interventional radiology, the risk of bowel ischemia was deemed to outweigh the potential benefit. Consequently, STS sclerotherapy was not performed.

Because TIPS was not feasible and targeted endovascular control could not be achieved, management was transitioned to medical therapy with octreotide and nonselective beta-blockade. Medical therapy consisted of octreotide 100 μg three times daily and carvedilol 3.125 mg twice daily. Carvedilol was selected as the initial nonselective beta-blocker in accordance with American Association for the Study of Liver Diseases guidance on the management of portal hypertension [13]. Ostomy bleeding subsequently decreased, and hemoglobin stabilized above 8 g/dL without further transfusion requirements. Because of persistent hypotension, carvedilol was transitioned to propranolol, with subsequent improvement in blood pressure.

The patient was discharged after 11 days of hospitalization. She was discharged with propranolol for a duration of 30 days and octreotide injections for a period of 7 days. She was scheduled for outpatient gastroenterology follow-up for management of ostomy bleeding and portal hypertension. Post-discharge care coordination notes documented continued clinical deterioration and a bedridden functional status, without documented recurrent ostomy bleeding. The patient died 15 days after discharge, precluding further outpatient follow-up.

## Discussion

This case demonstrates that peristomal varices may remain unrecognized despite repeated gastrointestinal bleeding evaluations, particularly when hemorrhage is presumed to originate from the bowel lumen [3-5]. This diagnostic challenge may be especially relevant in patients with advanced malignancy, who often have multiple competing sources of bleeding while simultaneously being at risk for portal hypertension from cirrhosis, hepatic metastases, or both. In our patient, portal hypertension was presumed to result from a combination of cirrhosis and extensive hepatic metastatic disease.

Computed tomographic angiography was performed twice with arterial and portal venous phase imaging, allowing characterization of the collateral anatomy. More importantly, reinterpretation of existing imaging proved more valuable than additional diagnostic testing once the clinical question shifted. Multidisciplinary review redirected the presumed bleeding source from the bowel lumen to peristomal varices and their associated portal venous collaterals, fundamentally changing management. Delayed recognition of peristomal varices can lead to recurrent hemorrhage, repeated transfusions, and unnecessary diagnostic evaluations. In our patient, the diagnosis was ultimately established through reinterpretation of previously obtained imaging rather than additional invasive testing, underscoring the value of multidisciplinary review when recurrent bleeding remains unexplained.

Consistent with previously reported cases, our patient presented with recurrent transfusion-dependent hemorrhage, with the diagnosis established only after careful reinterpretation of vascular imaging. In another case, a 65-year-old female with rectal carcinoma and Child-Pugh Class A liver disease presented with recurrent bleeding from her loop ileostomy. Previous investigations, including endoscopy and CT angiography, were unrevealing. Physical examination revealed obvious peristomal varices with bluish-purple discoloration, which supported the diagnosis. Venogram confirmed a definite network of peristomal collateral vessels between the superior mesenteric and inferior epigastric veins, which were most likely the source of bleeding [6]. The patient was successfully treated with embolization without recurrent bleeding, although the duration of follow-up was not reported. Our patient, in comparison, did not have obvious signs of peristomal varices on gross examination. Furthermore, the peristomal varices were small in caliber and could not be directly cannulated for intervention.

In another case, a 68-year-old female with stage IV colorectal adenocarcinoma metastatic to the liver, uterus, ovaries, lungs, and ureter underwent a palliative loop colostomy and subsequently developed recurrent stomal bleeding requiring repeated hospitalizations and blood transfusions. Bleeding was initially presumed to originate from the colon and treated conservatively with tranexamic acid, fresh frozen plasma, and packed red blood cell transfusions. Computed tomography later demonstrated peristomal varices supplied by the inferior mesenteric vein, and portal hypertension was attributed to compression of the right portal vein and thrombosis of the right and middle hepatic veins from metastatic liver invasion. Because TIPS was contraindicated by her oncologic disease and altered hepatic venous anatomy, she underwent successful percutaneous transhepatic coil embolization of the feeding varix with immediate cessation of bleeding and no recurrence at six months of follow-up [9]. In contrast, our patient's multifocal peristomal varices were too small to permit direct cannulation, and a retrograde portal venous approach was deferred because of concern for bowel ischemia, precluding definitive endovascular therapy.

In our patient, TIPS was considered prohibitively high risk because of advanced metastatic disease, poor functional status, prior hospice enrollment, and hemodynamic instability. STS sclerotherapy was also considered but ultimately deferred because of unfavorable vascular anatomy and concerns regarding bowel ischemia. However, unlike many published cases managed with TIPS, embolization, or percutaneous sclerotherapy, our patient's advanced metastatic malignancy, poor functional status, and unfavorable vascular anatomy precluded definitive intervention. This case therefore highlights the diagnostic and therapeutic challenges of peristomal variceal hemorrhage in patients for whom portal decompression and targeted endovascular therapies are not feasible. Consequently, medical management with octreotide and non-selective beta blockade represented the most appropriate therapeutic option.

Interpretation of this case is limited by several factors. Although CTA and portal venography demonstrated peristomal varices and their associated portal venous collaterals, no active extravasation was identified during imaging. Definitive treatment with TIPS or STS sclerotherapy could not be performed because of the patient's advanced malignancy, poor functional status, procedural risk, and unfavorable vascular anatomy. A tagged red blood cell scan might have provided additional localization during active bleeding but was not performed. Finally, the patient's death 15 days after discharge precluded assessment of long-term bleeding recurrence and durability of medical therapy.

## Conclusions

Peristomal varices should be considered in patients with recurrent ostomy bleeding and portal hypertension, particularly when standard gastrointestinal bleeding evaluations are unrevealing. Careful reassessment of cross-sectional imaging may identify portal venous collaterals and redirect management toward therapies that address the underlying portal hypertension.
